# Supplementary material for: The Comparison of Serum Exosome Protein Profile in Diagnosis of NSCLC Patients
Source: Int J Mol Sci. 2023 Sep 5;24(18):13669. doi: 10.3390/ijms241813669 (PMC10650331; doi:10.3390/ijms241813669)
Supplement: Supplementary file 1 [file ijms-24-13669-s001.zip › ijms-2578753-supplementary.pdf]

Table S1. Proteins identified by LC-MS analysis only in NSCLC patients, their molecular function and engagement in biological processes (based on UniProt database).

Molecular Function	Biological Process	Names	n	%
molecular transducer activity	nervous system process, signaling	Olfactory receptor 2AT4	12	80%
molecular transducer activity	nervous system process, signaling	Olfactory receptor 9G9	12	80%
DNA binding, transcription regulator activity	regulation of DNA-templated transcription, cell differentiation, anatomical structure development	Homeobox protein DBX1	11	73%
signaling receptor binding	anatomical structure development, signaling, cell differentiation	R-spondin-2	11	73%
molecular transducer activity	immune system process, signaling	Killer cell immunoglobulin-like receptor 2DL1	10	67%
signaling receptor binding	neuropeptide signaling pathway	Neurexophilin-3	10	67%
molecular transducer activity	nervous system process, signaling	Olfactory receptor 52R1	10	67%
oxidoreductase activity	immune system process	Gamma-interferon-inducible lysosomal thiol reductase	9	60%
molecular transducer activity	nervous system process, signaling	Olfactory receptor 13C4	9	60%
function unknown	biological process unknown	Protein FAM166B	9	60%
hydrolase activity	cell differentiation, anatomical structure development	Inactive phospholipid phosphatase 7	8	53%
DNA binding; protein domain specific binding	regulation of DNA-templated transcription	Myeloid leukemia factor 2	8	53%
molecular transducer activity	nervous system process, signaling	Olfactory receptor 2T4	8	53%
DNA binding, transporter activity, transcription regulator activity	anatomical structure development, regulation of DNA-templated transcription, cell differentiation, transmembrane transport	Protein odd-skipped-related 1	8	53%
carbohydrate binding	biological process unknown	Pulmonary surfactant-associated protein A2	8	53%
dna binding, transcription regulator activity	regulation of DNA-templated transcription, cell differentiation, anatomical structure development	Transcription factor JunB	8	53%
DNA and RNA binding, ranscription regulator activity	ribosome biogenesis, DNA-templated transcription	Activator of basal transcription 1	7	47%
hydrolase activity	lipid metabolic process,	Alkaline ceramidase 1	7	47%

lipid binding	biological process unknown	BPI fold-containing family A member 3	7	47%
hydrolase activity, catalytic activity, acting on a protein	proteolysis	Chymotrypsin-like elastase family member 2B	7	47%
function unknown	negative regulation of protein kinase activity, cell differentiation, anatomical structure development	Dysbindin domain-containing protein 2	7	47%
calcium ion sensor activity	cell differentiation, anatomical structure development	EF-hand domain-containing protein D1	7	47%
DNA binding	chromatin organization, regulation of DNA-templated transcription	GATA zinc finger domain-containing protein 1	7	47%
molecular transducer activity	immune system process	HLA class II histocompatibility antigen, DM alpha chain	7	47%
molecular transducer activity	immune system process	HLA class II histocompatibility antigen, DO beta chain	7	47%
DNA binding, transcription regulator activity	regulation of DNA-templated transcription, anatomical structure development	Homeobox protein Hox-C4	7	47%
GTPase activator activity	glutamine catabolic process, regulation of small GTPase mediated signal transduction	Inactive Rho GTPase-activating protein 11B	7	47%
function unknown	biological process unknown	Low-density lipoprotein receptor class A domain-containing protein 2	7	47%
lipid binding	vesicle-mediated transport, protein catabolic process	Multivesicular body subunit 12A	7	47%
function unknown	biological process unknown	Out at first protein homolog	7	47%
isomerase activity	carbohydrate metabolic process	Phosphoglycerate mutase 2	7	47%
DNA binding, transcription regulator activity	reproductive process, anatomical structure development	Putative transcription factor Ovo-like 1	7	47%
molecular adaptor activity	protein catabolic process, protein ubiquitination	SPRY domain-containing SOCS box protein 4	7	47%
oxidoreductase activity, protein kinase binding	anatomical structure development, cell differentiation, cellular response to oxidative stress	Thioredoxin-dependent peroxide reductase, mitochondrial	7	47%
DNA binding, transcription regulator activity	anatomical structure development, cell differentiation, regulation of DNA-templated transcription,	Transcription factor Ovo-like 2	7	47%

DNA binding, transcription regulator activity	regulation of DNA-templated transcription, immune system process, anatomical structure development, cell differentiation	Transcription factor PU.1	7	47%
function unknown	biological process unknown	Transmembrane protein 187	7	47%
function unknown	biological process unknown	Transmembrane protein 51	7	47%
spermatogenesis	reproductive process	Acrosomal protein SP-10	6	40%
DNA binding, transcription regulator activity	regulation of DNA-templated transcription, anatomical structure development, cell differentiation	GS homeobox 1	6	40%
function unknown	cell differentiation	Pleckstrin homology domain-containing family F member 1	6	40%
DNA binding, transcription regulator activity	regulation of DNA-templated transcription, anatomical structure development, cell differentiation	Protein lyl-1	6	40%
glutamate receptor binding, protein homodimerization activity	intracellular protein transport, vesicle-mediated transport, cell junction organization, anatomical structure development	Synapse differentiation-inducing gene protein 1	6	40%
structural molecule activity	epithelial cell differentiation, response to bacterium	Uroplakin-1b	6	40%
G protein activity	anatomical structure development, signaling, cell differentiation, cytoskeleton organization	Cdc42 effector protein 3	5	33%
glutamate receptor binding, protein homodimerization activity	intracellular protein transport, vesicle-mediated transport	Claudin-15	5	33%
RNA binding	biological process unknown	Embryonic polyadenylate-binding protein 2	5	33%
lipid binding, ubiquitin protein ligase binding	lipid metabolic process, protein catabolic process	Erlin-1	5	33%
GTPase activity	circulatory system process	GTP cyclohydrolase 1	5	33%
function unknown	biological process unknown	Inhibin beta C chain	5	33%
transferase activity, receptor ligand activity, atalytic activity, acting on a protein	immune system process, inflammatory response, anatomical structure development, cell differentiation	Interleukin-34	5	33%
function unknown	defense response to Gram-negative bacterium	Ly6/PLAUR domain-containing protein 8	5	33%

protein transporter activity	protein insertion into mitochondrial inner membrane	Mitochondrial import inner membrane translocase subunit Tim29	5	33%
hydrolase activity, catalytic activity, acting on a protein, molecular function regulator activity	negative regulation of peptidase activity	Peptidase inhibitor R3HDML	5	33%
isomerase activity, metal ion binding	carbohydrate metabolic process, protein glycosylation, nucleobase-containing small molecule metabolic proce	Phosphomannomutase 1	5	33%
function unknown	biological process unknown	Protein BEAN1	5	33%
function unknown	biological process unknown	Protein FAM210A	5	33%
function unknown	biological process unknown	Putative uncharacterized protein FLJ37218	5	33%
identical protein binding	signal transduction	Ras association domain-containing protein 3	5	33%
GTPase activity	intracellular signal transduction, protein localization to plasma membrane	Ras-related protein Rab-40A	5	33%
cytoskeletal protein binding	intracellular protein transport, nervous system process, membrane organization	Receptor expression-enhancing protein 2	5	33%
transferase activity	methylation	Thiol S-methyltransferase METTL7B	5	33%
hydrolase activity	lysosome organization, anatomical structure development, cell differentiation	Transmembrane protein 106B	5	33%
lyase activity	lipid metabolic process,	3-hydroxyacyl-CoA dehydratase 2	4	27%
receptor ligand activity	anatomical structure development, cell differentiation	Amphiregulin	4	27%
hydrolase activity, catalytic activity, acting on a protein	anatomical structure development, proteolysis	Cathepsin B	4	27%
molecular transducer activity	vesicle-mediated transport	Cation-dependent mannose-6-phosphate receptor	4	27%
transcription regulator activity, molecular function regulator activity	anatomical structure development, cell differentiation, reproductive process, regulation of DNA-templated transcription	Cbp/p300-interacting transactivator 2	4	27%
carbohydrate binding	vesicle-mediated transport, positive regulation of cytokine	C-type lectin domain family 9 member A	4	27%

	production, receptor-mediated endocytosis			
molecular transducer activity, molecular function regulator activity	signaling, Wnt signaling pathway	Dickkopf-related protein 2	4	27%
DNA binding, transcription regulator activity	anatomical structure development, cell differentiation, reproductive process, regulation of DNA-templated transcription	Homeobox protein BarH-like 1	4	27%
DNA binding, transcription regulator activity	anatomical structure development, cell differentiation, reproductive process, regulation of DNA-templated transcription	Homeobox protein MOX-1	4	27%
DNA binding, transcription regulator activity	anatomical structure development, cell differentiation, reproductive process, regulation of DNA-templated transcription	Homeobox protein notochord	4	27%
DNA binding, transcription regulator activity	anatomical structure development, cell differentiation, reproductive process, regulation of DNA-templated transcription	Intestine-specific homeobox	4	27%
identical protein binding, calcium-dependent protein binding	immune system process, defense response to other organism	Mannose-binding protein C	4	27%
transcription regulator activity	regulation of DNA-templated transcription, anatomical structure development	MyoD family inhibitor	4	27%
function unknown	biological process unknown	Proline-rich protein 23C	4	27%
isomerase activity, catalytic activity, acting on a protein	biological process unknown	Protein disulfide isomerase CRELD2	4	27%
function unknown	programmed cell death	Protein lifeguard 4	4	27%
function unknown	biological process unknown	Protein PBMUCL2	4	27%
function unknown	vesicle-mediated transport, protein maturation, membrane organization	Protein YIPF5	4	27%
transferase activity	methylation	Putative methyltransferase-like protein 7A	4	27%
transferase activity	catalytic activity, acting on a protein	Putative serine/threonine-protein kinase PRKY	4	27%
transferase activity, catalytic activity, acting on RNA	ribosome biogenesis, protein-containing complex assembly	rRNA methyltransferase 2, mitochondrial	4	27%

receptor ligand activity	immune system process, signaling, anatomical structure development	Secreted and transmembrane protein 1	4	27%
function unknown	biological process unknown	Surfeit locus protein 2	4	27%
mitochondrial proton-transporting ATP synthase complex binding	mitochondrion organization, protein-containing complex assembly	Transmembrane protein 70, mitochondrial	4	27%
lipid binding, hydrolase activity	lipid metabolic process,	Acyl-coenzyme A diphosphatase FITM2	3	20%
DNA binding	anatomical structure development	AN1-type zinc finger protein 5	3	20%
dynein heavy chain binding	biological process unknown	Axonemal dynein light intermediate polypeptide 1	3	20%
function unknown	biological process unknown	BRI3-binding protein	3	20%
function unknown	biological process unknown	Cell Division Cycle Associated 5	3	20%
hydrolase activity, catalytic activity, acting on a protein	regulation of DNA-templated transcription, anatomical structure development	Chymotrypsin-like elastase family member 1	3	20%
structural molecule activity	cell adhesion, anatomical structure development	Claudin-18	3	20%
carbohydrate binding, protein homodimerization activity	immune system process, defense response to other organism	C-type lectin domain family 2 member	3	20%
activin binding, fibronectin binding	regulation of DNA-templated transcription, anatomical structure development	Follistatin-related protein 3	3	20%
hydrolase activity	programmed cell death, immune system process	Granzyme A	3	20%
signaling (negative regulation of signal transduction by p53 class mediator, regulation of cellular response to stress)	biological process unknown	HUWE1 Associated Protein Modifying Stress Responses	3	20%
function unknown	biological process unknown	Keratin-associated protein 10-2	3	20%
function unknown	biological process unknown	Killer Cell Lectin Like Receptor C1	3	20%
function unknown	biological process unknown	Ly6/PLAUR domain-containing protein 3	3	20%
hydrolase activity	defense response to other organism	Lysozyme g-like protein 2	3	20%
lipid binding	membrane organization	Myelin peripheral protein	3	20%
function unknown	biological process unknown	PABIR family member 2	3	20%

transporter activity, ATP-dependent activity	programmed cell death, mitochondrion organization, signaling, transmembrane transport, membrane organization	Peptidyl-prolyl cis-trans isomerase F, mitochondrial	3	20%
function unknown	biological process unknown	Potassium Channel Tetramerization Domain Containing 2	3	20%
transferase activity, catalytic activity, acting on a protein	protein catabolic process	RING finger protein 175	3	20%
function unknown	biological process unknown	Serine protease 1	3	20%
function unknown	biological process unknown	Synapse differentiation-inducing gene protein 1-like	3	20%
function unknown	intracellular protein transport, vesicle-mediated transport	Transmembrane emp24 domain-containing protein 6	3	20%
transferase activity, ATP binding	nucleobase-containing small molecule metabolic process, carbohydrate derivative metabolic process	Adenylate kinase 2, mitochondrial	2	13%
function unknown	biological process unknown	ARL14 effector protein	2	13%
transferase activity, receptor ligand activity, molecular function regulator activity, catalytic activity, acting on a protein, transcription regulator activity	cell adhesion, immune system process, programmed cell death, signaling	CD40 ligand	2	13%
function unknown	cell adhesion	Claudin-12	2	13%
function unknown	biological process unknown	COMM domain-containing protein 5	2	13%
transferase activity	vitamin metabolic process	Corrinoid adenosyltransferase MMAB	2	13%
oxidoreductase activity, transferase activity	carbohydrate metabolic process, generation of precursor metabolites and energy	Cytochrome c oxidase assembly protein COX11, mitochondrial	2	13%
small molecule binding	biological process unknown	Epididymal-specific lipocalin-10	2	13%
function unknown	membrane organization	ER membrane protein complex subunit 3	2	13%
cytoskeletal protein binding	cytoskeleton organization, anatomical structure development	Four and a half LIM domains protein 3	2	13%
transferase activity	carbohydrate metabolic process, generation of precursor metabolites and energy	Glycogenin-1	2	13%

DNA binding, transcription regulator activity	regulation of DNA-templated transcription, anatomical structure development	Homeobox protein Nkx-2.8	2	13%
signaling receptor binding	signaling, anatomical structure development	Insulin-like growth factor-binding protein 1	2	13%
hydrolase activity, catalytic activity, acting on a protein	anatomical structure development, cell differentiation, nervous system process	Kallikrein-8	2	13%
function unknown	biological process unknown	Keratin-associated protein 10-8	2	13%
transferase activity	lipid metabolic process, membrane organization	Lactosylceramide 4-alpha-galactosyltransferase	2	13%
function unknown	biological process unknown	Ly6/PLAUR domain-containing protein 5	2	13%
function unknown	carbohydrate metabolic process, protein folding, protein glycosylation, lipid metabolic process	Mannose-P-dolichol utilization defect 1 protein	2	13%
function unknown	biological process unknown	Myb/SANT-like DNA-binding domain-containing protein 1	2	13%
receptor ligand activity	anatomical structure development, cell differentiation, signaling	Neuroblastoma suppressor of tumorigenicity 1	2	13%
molecular transducer activity	nervous system process, signaling	Olfactory receptor 12D2	2	13%
molecular transducer activity	nervous system process, signaling	Olfactory receptor 1E1	2	13%
molecular transducer activity	nervous system process, signaling	Olfactory receptor 2L2	2	13%
function unknown	biological process unknown	Potassium Channel Tetramerization Domain Containing 6	2	13%
function unknown	biological process unknown	Proteasome subunit alpha type-6	2	13%
receptor ligand activity, catalytic activity, acting on a protein, transcription regulator activity	anatomical structure development, signaling, cell differentiation	Protein Wnt-3a	2	13%
function unknown	vesicle-mediated transport, membrane organization	Protein YIPF4	2	13%
hydrolase activity	lipid metabolic process, sulfur compound metabolic process	Putative cytosolic acyl coenzyme A thioester hydrolase-like	2	13%
GTPase activity, molecular function regulator activity	regulation of DNA-templated transcription	Ras-like protein family member 11A	2	13%

function unknown	biological process unknown	Regulatory Factor X Associated Ankyrin Containing Protein	2	13%
function unknown	biological process unknown	Ring Finger Protein 125	2	13%
function unknown	biological process unknown	RINGO Cell Cycle Regulator Family Member E4	2	13%
function unknown	vesicle-mediated transport, reproductive process	Sperm acrosome membrane-associated protein 3	2	13%
DNA binding	DNA repair, telomere organization	TCF3 fusion partner	2	13%
function unknown	biological process unknown	Tetraspanin-3	2	13%
transferase activity, catalytic activity, acting on a protein	protein phosphorylation	Tribbles Pseudokinase 2	2	13%
function unknown	biological process unknown	Uncharacterized protein C17orf50	2	13%
function unknown	biological process unknown	Uncharacterized protein C22orf46	2	13%
receptor ligand activity	anatomical structure development, signaling, cell differentiation, cell motility, immune system process	Vascular endothelial growth factor D	2	13%
function unknown	biological process unknown	Zinc finger FYVE domain-containing protein 21	2	13%

Table S2. Proteins identified by LC-MS analysis only in control group, their molecular function and engagement in biological processes (based on UniProt database).

Molecular Function	Biological Process	Names	n	%
molecular transducer activity	nervous system process, signaling	Olfactory receptor 14I1	13	81%
molecular transducer activity	nervous system process, signaling	Putative olfactory receptor 52L2	12	75%
transporter activity	transmembrane transport	Aquaporin-12A	7	44%
molecular transducer activity	nervous system process, signaling	Olfactory receptor 8B8	7	44%
molecular transducer activity	nervous system process, signaling	Olfactory receptor 52I1	7	44%
molecular transducer activity	nervous system process, signaling	Olfactory receptor 2AG1	6	38%
function unknown	biological process unknown	Putative uncharacterized protein encoded by LINC01599	6	38%
DNA binding, transcription regulator activity	regulation of DNA-templated transcription, anatomical structure development	Homeobox protein Hox-C13	5	31%
molecular transducer activity	nervous system process, signaling	Olfactory receptor 10P1	5	31%
molecular transducer activity	nervous system process, signaling	Olfactory receptor 2M4	5	31%
function unknown	biological process unknown	Signal Sequence Receptor Subunit 2	5	31%
lipid binding, transferase activity	lipid metabolic process, vitamin metabolic process	Lecithin retinol acyltransferase	5	31%
lipid binding	cell adhesion, myofibroblast contraction	Mammalian ependymin-related protein 1	5	31%
GTPase activity	vesicle-mediated transport,	Ras-related protein Rab-33B	5	31%
DNA binding, transcription regulator activity	regulation of DNA-templated transcription, cell differentiation, anatomical structure development, nervous system process	Neural retina-specific leucine zipper protein	5	31%
DNA binding, transcription regulator activity	regulation of DNA-templated transcription, immune system process, anatomical structure development, cell differentiation	BarH-like 1 homeobox protein	4	25%

molecular carrier activity	mitochondrion organization, intracellular protein transport, programmed cell death, cell differentiation, anatomical structure development	Mitochondrial carrier homolog 2	4	25%
transferase activity	protein catabolic process, immune system process,	Ubiquitin-conjugating enzyme E2 J1	4	25%
molecular transducer activity	nervous system process, signaling	Olfactory receptor 52B4	4	25%
molecular transducer activity	nervous system process, signaling	Olfactory receptor 52B2	4	25%
function unknown	immune system process	CD48 antigen	4	25%
transcription coregulator activity	Transcription regulation	Mediator of RNA polymerase II transcription subunit 19	4	25%
cholesterol binding	Lipid transport	StAR-related lipid transfer protein 4	4	25%
oxidoreductase activity	Ubiquinone biosynthesis	5-demethoxyubiquinone hydroxylase, mitochondrial	4	25%
function unknown	positive regulation of neuron differentiation,	Brorin	3	19%
molecular transducer activity	nervous system process, signaling	Olfactory receptor 5AC1	3	19%
molecular transducer activity	nervous system process, signaling	Olfactory receptor 10Z1	3	19%
hydrolase activit, catalytic activity, acting on a protein	proteolysis	Tryptase gamma	3	19%
molecular transducer activity	nervous system process, signaling	Olfactory receptor 2H2	3	19%
hydrolase activity, molecular transducer activity, catalytic activity, acting on a protein	wound healing, cell adhesion, signaling, cell motility, protein maturation	Urokinase plasminogen activator surface receptor	3	19%
function unknown	immune system process, cytoskeleton organization, cell differentiation, anatomical structure development	Sialic acid-binding Ig-like lectin 15	3	19%
molecular transducer activity	nervous system process, signaling	Olfactory receptor 8U1	3	19%
structural molecule activity, lipid binding,	nucleocytoplasmic transport, intracellular protein transport	Nucleoporin NUP35	3	19%
protein tyrosine kinase binding	protein localization	Phytanoyl-CoA hydroxylase-interacting protein	3	19%

transferase activity	cellular modified amino acid metabolic process, muscle system process, reproductive process, anatomical structure development	Guanidinoacetate N-methyltransferase	3	19%
hydrolase activity, catalytic activity, acting on a protein	cell motility, proteolysis	Putative trypsin-6	3	19%
function unknown	signaling, immune system process, defense response to other organism	Tetraspanin-6	3	19%
function unknown	biological process unknown	Tektin bundle interacting protein 1	3	19%
function unknown	biological process unknown	Cation channel sperm-associated auxiliary subunit TMEM249	3	19%
hydrolase activity,	protein glycosylation, nucleobase-containing small molecule metabolic process, carbohydrate derivative metabolic process	Uridine diphosphate glucose pyrophosphatase NUDT14	3	19%
identical protein binding	signaling, cell differentiation	Complement C1q-like protein 4	3	19%
molecular transducer activity, molecular function regulator activity	immune system process	Interleukin-18-binding protein	3	19%
transferase activity, catalytic activity, acting on a protein	protein polyubiquitination, mitotic cell cycle	Ubiquitin-conjugating enzyme E2 E2	3	19%
receptor ligand activity		Insulin, isoform 2	3	19%
function unknown	biological process unknown	Cilia- and flagella-associated protein 299	3	19%
transferase activity	nucleobase-containing small molecule metabolic process, carbohydrate derivative metabolic process	GTP:AMP phosphotransferase AK3, mitochondrial	3	19%
function unknown	biological process unknown	Transmembrane protein 247	3	19%
function unknown	intracellular protein transport, vesicle-mediated transport	Putative uncharacterized protein encoded by LINC00869	2	13%
molecular transducer activity	nervous system process, signaling	Olfactory receptor 6C3	2	13%
molecular transducer activity	immune system process, signaling, inflammatory response, cell adhesion	Interleukin-20 receptor subunit beta	2	13%
molecular transducer activity	nervous system process, signaling	Olfactory receptor 1M1	2	13%

oxidoreductase activity	lipid metabolic process,	Phytanoyl-CoA dioxygenase, peroxisomal	2	13%
transferase activity, catalytic activity, acting on a protein	post-translational protein modification, phosphorylation	Ketosamine-3-kinase	2	13%
molecular transducer activity	signaling	Mas-related G-protein coupled receptor member G	2	13%
structural molecule activity, cytoskeletal protein binding	cytoskeleton organization, protein-containing complex assembly,	Actin-related protein 2/3 complex subunit 2	2	13%
DNA binding, transcription regulator activity	regulation of DNA-templated transcription, signaling	Krueppel-like factor 14	2	13%
GTPase activity, transferase activity, molecular function regulator activity, catalytic activity, acting on a protein	carbohydrate metabolic process, cytoskeleton organization, signaling	Target of rapamycin complex subunit LST8	2	13%
DNA binding, transcription regulator activity	regulation of DNA-templated transcription, signaling, cell differentiation, anatomical structure development	Transcription factor ATOH8	2	13%
hydrolase activity, catalytic activity, acting on a protein	anatomical structure development, cell differentiation, protein catabolic process, extracellular matrix organization	Cathepsin K	2	13%
oxidoreductase activity	lipid metabolic process, immune system process	Cholesterol 25-hydroxylase	2	13%
hydrolase activity	lipid metabolic process	Acyl-coenzyme A thioesterase THEM5	2	13%
histone binding, transcription regulator activity	regulation of DNA-templated transcription, programmed cell death	Inhibitor of growth protein 4	2	13%
transferase activity, molecular transducer activity, molecular function regulator activity, catalytic activity, acting on a protein	immune system process, signaling, cell differentiation, anatomical structure development	B-cell antigen receptor complex-associated protein alpha chain	2	13%
transcription regulator activity,	immune system process, defense response to other organism, signaling	C-type lectin domain family 7 member A	2	13%
transcription regulator activity, general transcription initiation factor activity	regulation of DNA-templated transcription, programmed cell death	Transcription initiation factor TFIID subunit 9B	2	13%
signaling receptor binding	signaling, muscle system process	Neuromedin-U	2	13%

GTPase activity, transferase activity, molecular function regulator activity, catalytic activity, acting on a protein	immune system process, cytoskeleton organization, cell adhesion, vesicle-mediated transport, signaling, anatomical structure development, cell motility	Ras-related C3 botulinum toxin substrate 2	2	13%
GTPase activity, transferase activity	cytoskeleton organization, signaling, cell differentiation, cell junction organization, anatomical structure development	Cell division control protein 42 homolog	2	13%
transferase activity, catalytic activity, acting on a protein	chromatin organization, lipid metabolic process	N-alpha-acetyltransferase 40	2	13%
function unknown	biological process unknown	UPF0688 protein C1orf174	2	13%
DNA binding, transcription regulator activity	regulation of DNA-templated transcription, cell differentiation, anatomical structure development	Forkhead box protein L3	2	13%
function unknown	biological process unknown	Membrane protein FAM174A	2	13%
function unknown	biological process unknown	Uncharacterized protein encoded by LINC01547	2	13%
identical protein binding, metal ion binding	chromatin organization, chromosome segregation, protein-containing complex assembly	Protein Mis18-alpha	2	13%
structural molecule activity, cytoskeletal protein binding	cytoskeleton organization, cell differentiation, anatomical structure development	Cysteine and glycine-rich protein 2	2	13%
function unknown	intracellular protein transport, protein maturation	Signal peptidase complex subunit 2	2	13%
mitochondrial ribosome binding	mitochondrion organization, anatomical structure development, protein-containing complex assembly	Transmembrane protein 223	2	13%
function unknown	biological process unknown	DnaJ homolog subfamily C member 5B	2	13%
protein-containing complex binding	protein catabolic process, protein-containing complex assembly	BTB/POZ domain-containing protein KCTD5	2	13%
transferase activity, catalytic activity, acting on a protein	immune system process, defense response to other organism, autophagy, protein catabolic process	3 ubiquitin-protein ligase RNF166	2	13%

transferase activity, catalytic activity, acting on a protein, receptor ligand activity	anatomical structure development, cell motility, signaling, cell differentiation, regulation of DNA-templated transcription	Platelet-derived growth factor subunit B	2	13%
function unknown	DNA replication, DNA repair, DNA recombination chromatin organization	Chromatin modification-related protein MEAF6	2	13%
function unknown	vesicle-mediated transport, intracellular protein transport	Coatomer subunit zeta-2	2	13%
protein-containing complex binding	programmed cell death, signaling, reproductive process, mitochondrion organization	Bcl-2-like protein 2	2	13%
virus receptor activity, lipid binding, transferase activity	immune system process, signaling, cell differentiation, protein catabolic process, anatomical structure development, cell adhesion	CD81 antigen	2	13%
DNA binding, transcription regulator activity	regulation of DNA-templated transcription	Putative homeobox protein NANOG2	2	13%

Table S3. Enrichment analysis of protein hub clusters.

#category	term ID	term description	p value
		**Control clusters**	
		**CD48 MLST8 RAC2**	
KEGG	hsa04650	Natural killer cell mediated cytotoxicity	0.0393
		**CD81 CDC42 PLAUR**	
GO Process	GO:0022409	Positive regulation of cell-cell adhesion	0.0353
GO Component	GO:0005925	Focal adhesion	0.0153
COMPARTMENTS	GOCC:0005925	Focal adhesion	0.0052
		**PHYH PHYHIP**	
DISEASES	DOID:10582	Refsum disease	0.00023
		**SPCS2 SSR2**	
STRING clusters	CL:3273	Protein export, and Translocon-associated	0.0041
		**ARPC2 RAB33B**	
no significant enrichment detected			
		**IL20RB PDGFB**	
KEGG	hsa04630	JAK-STAT signaling pathway	0.0229
		**LRAT NRL**	
Monarch HPO	HP:0000533	Chorioretinal atrophy	0.0210
Monarch HPO	HP:0000550	Undetectable electroretinogram	0.0210
Monarch HPO	HP:0000608	Macular degeneration	0.0369
Monarch HPO	HP:0007675	Progressive night blindness	0.0397
Monarch HPO	HP:0000563	Keratoconus	0.0462
Monarch HPO	HP:0000842	Hyperinsulinemia	0.0462
Monarch HPO	HP:0000980	Pallor	0.0462
Monarch HPO	HP:0000987	Atypical scarring of skin	0.0462
Monarch HPO	HP:0001105	Retinal atrophy	0.0462
Monarch HPO	HP:0001133	Constriction of peripheral visual field	0.0462
Monarch HPO	HP:0005978	Type II diabetes mellitus	0.0462
Monarch HPO	HP:0008046	Abnormal retinal vascular morphology	0.0462
Monarch HPO	HP:0031605	Abnormality of fundus pigmentation	0.0462
Monarch HPO	HP:0000602	Ophthalmoplegia	0.0481
Monarch HPO	HP:0000510	Rod-cone dystrophy	0.0484
Monarch HPO	HP:0000613	Photophobia	0.0484
Monarch HPO	HP:0007703	Abnormality of retinal pigmentation	0.0484
UniProt Keywords	KW-0844	Vision	0.0240

**TAF9B TSPAN6**			
no significant enrichment detected			
**ING4 MEAF6**			
GO Process	GO:0043983	Histone h4-k12 acetylation	0.0024
GO Process	GO:0043981	Histone h4-k5 acetylation	0.0051
GO Process	GO:0043982	Histone h4-k8 acetylation	0.0051
GO Process	GO:0043966	Histone h3 acetylation	0.0181
GO Component	GO:0000123	Histone acetyltransferase complex	0.0317
COMPARTMENTS	GOCC:0070776	MOZ/MORF histone acetyltransferase complex	0.00054
**UBE2E2 UBE2J1**			
GO Function	GO:0061631	Ubiquitin conjugating enzyme activity	0.0146
STRING clusters	CL:2881	Ubiquitin-conjugating enzyme E2, catalytic domain homologues, and FAT10 activating enzyme activity	0.0025
KEGG	hsa04120	Ubiquitin mediated proteolysis	0.0164
Pfam	PF00179	Ubiquitin-conjugating enzyme	0.0148
InterPro	IPR000608	Ubiquitin-conjugating enzyme E2	0.0276
InterPro	IPR016135	Ubiquitin-conjugating enzyme/RWD-like	0.0276
**NSCLC clusters**			
**BATF CD40LG IL34 LYL1 SPI1**			
GO Process	GO:0030098	Lymphocyte differentiation	0.0015
GO Process	GO:0002320	Lymphoid progenitor cell differentiation	0.0127
GO Process	GO:0019221	Cytokine-mediated signaling pathway	0.0127
GO Process	GO:0042113	B cell activation	0.0127
GO Process	GO:0043011	Myeloid dendritic cell differentiation	0.0127
GO Process	GO:0045190	Isotype switching	0.0141
GO Process	GO:0010628	Positive regulation of gene expression	0.0194
GO Process	GO:0002376	Immune system process	0.0202
WikiPathways	WP3995	Prion disease pathway	0.0198
WikiPathways	WP2849	Hematopoietic stem cell differentiation	0.0271
**HLA-DMA HLA-DOB IFI30 RFXANK**			
GO Process	GO:0019886	Antigen processing and presentation of exogenous peptide antigen via mhc class ii	0.0064
GO Function	GO:0023026	MHC class II protein complex binding	0.0155
GO Component	GO:0042613	MHC class II protein complex	0.0064
STRING clusters	CL:19310	Mixed, incl. mhc class ii protein complex, and mhc class ii deficiency	0.00012
STRING clusters	CL:19314	MHC II	0.0047

KEGG	hsa04612	Antigen processing and presentation	4.22e-08
KEGG	hsa05152	Tuberculosis	0.00044
KEGG	hsa05310	Asthma	0.0014
KEGG	hsa04672	Intestinal immune network for IgA production	0.0017
KEGG	hsa04940	Type I diabetes mellitus	0.0017
KEGG	hsa05320	Autoimmune thyroid disease	0.0017
KEGG	hsa05330	Allograft rejection	0.0017
KEGG	hsa05332	Graft-versus-host disease	0.0017
KEGG	hsa05416	Viral myocarditis	0.0019
KEGG	hsa05321	Inflammatory bowel disease	0.0020
KEGG	hsa05140	Leishmaniasis	0.0024
KEGG	hsa04640	Hematopoietic cell lineage	0.0033
KEGG	hsa04658	Th1 and Th2 cell differentiation	0.0033
KEGG	hsa04659	Th17 cell differentiation	0.0033
KEGG	hsa05145	Toxoplasmosis	0.0033
KEGG	hsa05150	Staphylococcus aureus infection	0.0033
KEGG	hsa05322	Systemic lupus erythematosus	0.0033
KEGG	hsa05323	Rheumatoid arthritis	0.0033
KEGG	hsa04514	Cell adhesion molecules	0.0053
KEGG	hsa04145	Phagosome	0.0054
KEGG	hsa05164	Influenza A	0.0069
KEGG	hsa05169	Epstein-Barr virus infection	0.0089
KEGG	hsa05166	Human T-cell leukemia virus 1 infection	0.0102
KEGG	hsa05168	Herpes simplex virus 1 infection	0.0490
Reactome	HSA-2132295	MHC class II antigen presentation	0.0021
COMPARTMENTS	GOCC:0042613	MHC class II protein complex	0.0087
UniProt Keywords	KW-0491	MHC II	0.0017
UniProt Keywords	KW-0458	Lysosome	0.0055
UniProt Keywords	KW-0391	Immunity	0.0170
InterPro	IPR014745	MHC class II, alpha/beta chain, N-terminal	0.0288
SMART	SM00407	Immunoglobulin C-Type	0.0177
		**COX11 PPIF PRDX3**	
UniProt Keywords	KW-0809	Transit peptide	0.0146
		**ABT1 FTSJ2 PSMA6**	
no significant enrichment detected			
		**ACRV1 LYPD3 SPACA3**	
InterPro	IPR016054	Ly-6 antigen/uPA receptor-like	0.0268

**EMC3 ERLIN1 REEP2**			
UniProt Keywords	KW-0890	Hereditary spastic paraplegia	0.0187
**GZMA KIR2DL1 KLRC1**			
STRING clusters	CL:19097	MHC class I protein complex binding, and MHC_I C-terminus	0.0026
KEGG	hsa05332	Graft-versus-host disease	0.0037
KEGG	hsa04612	Antigen processing and presentation	0.0055
KEGG	hsa04650	Natural killer cell mediated cytotoxicity	0.0131
**DKK2 RSPO2 WNT3A**			
GO Process	GO:0090263	Positive regulation of canonical wnt signaling pathway	0.0058
GO Process	GO:0016055	Wnt signaling pathway	0.0189
GO Process	GO:0071542	Dopaminergic neuron differentiation	0.0189
GO Function	GO:0039706	Co-receptor binding	0.0053
STRING clusters	CL:21040	Wnt signaling pathway, and beta-catenin-TCF complex assembly	0.00061
STRING clusters	CL:21045	Wnt signaling in kidney disease, and Negative regulation of TCF-dependent signaling by WNT ligand antagonists	0.0267
KEGG	hsa04310	Wnt signaling pathway	0.00017
Reactome	HSA-201681	TCF dependent signaling in response to WNT	0.0024
Reactome	HSA-3772470	Negative regulation of TCF-dependent signaling by WNT ligand antagonists	0.0024
Reactome	HSA-4641263	Regulation of FZD by ubiquitination	0.0026
Reactome	HSA-4791275	Signaling by WNT in cancer	0.0043
WikiPathways	WP4258	lncRNA in canonical Wnt signaling and colorectal cancer	0.0396
WikiPathways	WP428	Wnt signaling	0.0396
WikiPathways	WP4336	ncRNAs involved in Wnt signaling in hepatocellular carcinoma	0.0396
UniProt Keywords	KW-0879	Wnt signaling pathway	0.00068
UniProt Keywords	KW-0217	Developmental protein	0.0393
**COMMD5 KCTD6 SPSB4**			
Reactome	HSA-8951664	Neddylation	0.0036
UniProt Keywords	KW-0833	Ubl conjugation pathway	0.0299
**CLDN12 CLDN15**			
GO Process	GO:0016338	Calcium-independent cell-cell adhesion via plasma membrane cell-adhesion molecules	0.0170
GO Component	GO:0016328	Lateral plasma membrane	0.0173
GO Component	GO:0005923	Bicellular tight junction	0.0338

STRING clusters	CL:22160	Calcium-independent cell-cell adhesion via plasma membrane cell-adhesion molecules, and tight junction protein zo	0.0045
Reactome	HSA-420029	Tight junction interactions	0.0056
WikiPathways	WP4239	Epithelial to mesenchymal transition in colorectal cancer	0.0462
COMPARTMENTS	GOCC:0005923	Bicellular tight junction	0.0353
UniProt Keywords	KW-0796	Tight junction	0.0168
InterPro	IPR017974	Claudin, conserved site	0.0098
**LYPD5 LYPD8**			
UniProt Keywords	KW-0336	GPI-anchor	0.0344
Pfam	PF00021	u-PAR/Ly-6 domain	0.0038
InterPro	IPR016054	Ly-6 antigen/uPA receptor-like	0.0089
**CTSB M6PR**			
KEGG	hsa04142	Lysosome	0.0143

Table S4. Frequency of proteins identified by LC-MS analysis only in NSCLC patients according to histopathological subtypes of tumor.

**Protein name**	**Patients with diagnosed AC subtype**	**Patients with diagnosed SCC subtype**
Olfactory receptor 2AT4	75%	83%
Olfactory receptor 9G9	75%	83%
Homeobox protein DBX1	75%	67%
R-spondin-2	75%	67%
Killer cell immunoglobulin-like receptor 2DL1	63%	67%
Neurexophilin-3	50%	83%
Olfactory receptor 52R1	63%	67%
Gamma-interferon-inducible lysosomal thiol reductase	50%	67%
Olfactory receptor 13C4	75%	50%

Protein FAM166B	75%	50%
Inactive phospholipid phosphatase 7	50%	50%
Myeloid leukemia factor 2	50%	50%
Olfactory receptor 2T4	50%	50%
Protein odd-skipped-related 1	50%	50%
Pulmonary surfactant-associated protein A2	50%	50%
Transcription factor JunB	63%	50%
Activator of basal transcription 1	38%	50%
Alkaline ceramidase 1	38%	50%
BPI fold-containing family A member 3	38%	50%
Chymotrypsin-like elastase family member 2B	38%	50%
Dysbindin domain-containing protein 2	38%	50%
EF-hand domain-containing protein D1	50%	50%
GATA zinc finger domain-containing protein 1	38%	50%
HLA class II histocompatibility antigen, DM alpha chain	38%	50%
HLA class II histocompatibility antigen, DO beta chain	38%	50%
Homeobox protein Hox-C4	38%	50%
Inactive Rho GTPase-activating protein 11B	38%	50%
Low-density lipoprotein receptor class A domain-containing protein 2	38%	50%
Multivesicular body subunit 12A	38%	50%
Out at first protein homolog	38%	50%
Phosphoglycerate mutase 2	50%	50%
Putative transcription factor Ovo-like 1	38%	50%
SPRY domain-containing SOCS box protein 4	38%	50%
Thioredoxin-dependent peroxide reductase, mitochondrial	38%	50%
Transcription factor Ovo-like 2	38%	50%
Transcription factor PU.1	38%	50%
Transmembrane protein 187	38%	50%
Transmembrane protein 51	38%	50%
Acrosomal protein SP-10	38%	33%
GS homeobox 1	38%	50%
Pleckstrin homology domain-containing family F member 1	38%	33%
Protein lyl-1	25%	50%

Synapse differentiation-inducing gene protein 1	38%	33%
Uroplakin-1b	38%	50%
Cdc42 effector protein 3	38%	33%
Claudin-15	25%	33%
Embryonic polyadenylate-binding protein 2	25%	50%
Erlin-1	25%	33%
GTP cyclohydrolase 1	25%	33%
Inhibin beta C chain	25%	33%
Interleukin-34	38%	33%
Ly6/PLAUR domain-containing protein 8	25%	33%
Mitochondrial import inner membrane translocase subunit Tim29	38%	17%
Peptidase inhibitor R3HDML	25%	33%
Phosphomannomutase 1	25%	33%
Protein BEAN1	38%	17%
Protein FAM210A	25%	33%
Putative uncharacterized protein FLJ37218	50%	17%
Ras association domain-containing protein 3	38%	33%
Ras-related protein Rab-40A	25%	33%
Receptor expression-enhancing protein 2	25%	33%
Thiol S-methyltransferase METTL7B	38%	33%
Transmembrane protein 106B	38%	33%
3-hydroxyacyl-CoA dehydratase 2	25%	33%
Amphiregulin	25%	17%
Cathepsin B	13%	33%
Cation-dependent mannose-6-phosphate receptor	13%	33%
Cbp/p300-interacting transactivator 2	38%	17%
C-type lectin domain family 9 member A	38%	17%
Dickkopf-related protein 2	25%	17%
Homeobox protein BarH-like 1	25%	33%
Homeobox protein MOX-1	25%	17%
Homeobox protein notochord	25%	17%
Intestine-specific homeobox	25%	17%
Mannose-binding protein C	25%	33%

MyoD family inhibitor	38%	17%
Proline-rich protein 23C	25%	17%
Protein disulfide isomerase CRELD2	13%	33%
Protein lifeguard 4	13%	33%
Protein PBMUCL2	25%	17%
Protein YIPF5	25%	17%
Putative methyltransferase-like protein 7A	25%	17%
Putative serine/threonine-protein kinase PRKY	25%	33%
rRNA methyltransferase 2, mitochondrial	25%	33%
Secreted and transmembrane protein 1	25%	17%
Surfeit locus protein 2	38%	0%
Transmembrane protein 70, mitochondrial	25%	17%
Acyl-coenzyme A diphosphatase FITM2	13%	17%
AN1-type zinc finger protein 5	13%	33%
Axonemal dynein light intermediate polypeptide 1	25%	17%
BRI3-binding protein	13%	33%
Cell Division Cycle Associated 5	13%	33%
Chymotrypsin-like elastase family member 1	25%	17%
Claudin-18	13%	33%
C-type lectin domain family 2 member	13%	33%
Follistatin-related protein 3	25%	17%
Granzyme A	13%	17%
HUWE1 Associated Protein Modifying Stress Responses	38%	0%
Keratin-associated protein 10-2	13%	17%
Killer Cell Lectin Like Receptor C1	13%	17%
Ly6/PLAUR domain-containing protein 3	25%	0%
Lysozyme g-like protein 2	13%	17%
Myelin peripheral protein	13%	33%
PABIR family member 2	13%	33%
Peptidyl-prolyl cis-trans isomerase F, mitochondrial	0%	33%
Potassium Channel Tetramerization Domain Containing 2	25%	17%
RING finger protein 175	25%	17%
Serine protease 1	13%	17%

Synapse differentiation-inducing gene protein 1-like	13%	17%
Transmembrane emp24 domain-containing protein 6	13%	17%
Adenylate kinase 2, mitochondrial	25%	0%
ARL14 effector protein	13%	17%
CD40 ligand	13%	17%
Claudin-12	13%	17%
COMM domain-containing protein 5	0%	33%
Corrinoid adenosyltransferase MMAB	13%	17%
Cytochrome c oxidase assembly protein COX11, mitochondrial	25%	0%
Epididymal-specific lipocalin-10	13%	17%
ER membrane protein complex subunit 3	13%	17%
Four and a half LIM domains protein 3	13%	17%
Glycogenin-1	13%	17%
Homeobox protein Nkx-2.8	25%	0%
Insulin-like growth factor-binding protein 1	25%	0%
Kallikrein-8	25%	0%
Keratin-associated protein 10-8	13%	17%
Lactosylceramide 4-alpha-galactosyltransferase	0%	33%
Ly6/PLAUR domain-containing protein 5	0%	17%
Mannose-P-dolichol utilization defect 1 protein	13%	17%
Myb/SANT-like DNA-binding domain-containing protein 1	13%	17%
Neuroblastoma suppressor of tumorigenicity 1	25%	0%
Olfactory receptor 12D2	13%	17%
Olfactory receptor 1E1	13%	17%
Olfactory receptor 2L2	13%	17%
Potassium Channel Tetramerization Domain Containing 6	25%	0%
Proteasome subunit alpha type-6	13%	17%
Protein Wnt-3a	0%	17%
Protein YIPF4	13%	17%
Putative cytosolic acyl coenzyme A thioester hydrolase-like	13%	17%
Ras-like protein family member 11A	13%	17%
Regulatory Factor X Associated Ankyrin Containing Protein	13%	17%
Ring Finger Protein 125	13%	17%

RINGO Cell Cycle Regulator Family Member E4	13%	17%
Sperm acrosome membrane-associated protein 3	0%	33%
TCF3 fusion partner	0%	33%
Tetraspanin-3	13%	17%
Tribbles Pseudokinase 2	13%	17%
Uncharacterized protein C17orf50	0%	17%
Uncharacterized protein C22orf46	13%	17%
Vascular endothelial growth factor D	0%	33%
Zinc finger FYVE domain-containing protein 21	25%	0%

Table S5. Frequency of proteins identified by LC-MS analysis only in NSCLC patients according to T feature (pTNM classification).

**Protein name**	**NSCLC patients with T1 feature[a]**	**NSCLC patients with T2 feature[b]**	**NSCLC patients with T3+T4 feature[c]**
Olfactory receptor 2AT4	100%	80%	50%
Olfactory receptor 9G9	83%	100%	50%
Homeobox protein DBX1	100%	60%	50%
R-spondin-2	83%	80%	50%
Killer cell immunoglobulin-like receptor 2DL1	83%	60%	50%
Neurexophilin-3	67%	60%	75%
Olfactory receptor 52R1	83%	80%	25%
Gamma-interferon-inducible lysosomal thiol reductase	67%	60%	50%

Olfactory receptor 13C4	83%	60%	25%
Protein FAM166B	50%	80%	50%
Inactive phospholipid phosphatase 7	83%	60%	0%
Myeloid leukemia factor 2	67%	60%	25%
Olfactory receptor 2T4	67%	60%	25%
Protein odd-skipped-related 1	67%	60%	25%
Pulmonary surfactant-associated protein A2	67%	60%	25%
Transcription factor JunB	67%	60%	25%
Activator of basal transcription 1	67%	60%	0%
Alkaline ceramidase 1	67%	60%	0%
BPI fold-containing family A member 3	67%	60%	0%
Chymotrypsin-like elastase family member 2B	67%	60%	0%
Dysbindin domain-containing protein 2	67%	60%	0%
EF-hand domain-containing protein D1	50%	60%	25%
GATA zinc finger domain-containing protein 1	67%	60%	0%
HLA class II histocompatibility antigen, DM alpha chain	67%	60%	0%
HLA class II histocompatibility antigen, DO beta chain	67%	60%	0%
Homeobox protein Hox-C4	67%	60%	0%
Inactive Rho GTPase-activating protein 11B	67%	60%	0%
Low-density lipoprotein receptor class A domain-containing protein 2	67%	60%	0%
Multivesicular body subunit 12A	67%	60%	0%
Out at first protein homolog	67%	60%	0%
Phosphoglycerate mutase 2	50%	40%	50%
Putative transcription factor Ovo-like 1	67%	60%	0%
SPRY domain-containing SOCS box protein 4	67%	60%	0%
Thioredoxin-dependent peroxide reductase, mitochondrial	67%	60%	0%
Transcription factor Ovo-like 2	67%	60%	0%
Transcription factor PU.1	67%	60%	0%
Transmembrane protein 187	67%	60%	0%
Transmembrane protein 51	50%	60%	25%
Acrosomal protein SP-10	50%	60%	0%
GS homeobox 1	50%	60%	0%
Pleckstrin homology domain-containing family F member 1	67%	40%	0%

Protein lyl-1	67%	40%	0%
Synapse differentiation-inducing gene protein 1	50%	40%	25%
Uroplakin-1b	50%	60%	0%
Cdc42 effector protein 3	50%	20%	25%
Claudin-15	33%	40%	25%
Embryonic polyadenylate-binding protein 2	50%	40%	0%
Erlin-1	33%	40%	25%
GTP cyclohydrolase 1	50%	20%	25%
Inhibin beta C chain	33%	40%	25%
Interleukin-34	33%	40%	25%
Ly6/PLAUR domain-containing protein 8	33%	60%	0%
Mitochondrial import inner membrane translocase subunit Tim29	50%	40%	0%
Peptidase inhibitor R3HDML	33%	40%	25%
Phosphomannomutase 1	33%	60%	0%
Protein BEAN1	50%	40%	0%
Protein FAM210A	33%	60%	0%
Putative uncharacterized protein FLJ37218	17%	40%	50%
Ras association domain-containing protein 3	33%	40%	25%
Ras-related protein Rab-40A	33%	40%	25%
Receptor expression-enhancing protein 2	33%	40%	25%
Thiol S-methyltransferase METTL7B	33%	40%	25%
Transmembrane protein 106B	33%	60%	0%
3-hydroxyacyl-CoA dehydratase 2	33%	20%	25%
Amphiregulin	33%	40%	0%
Cathepsin B	33%	20%	25%
Cation-dependent mannose-6-phosphate receptor	67%	0%	0%
Cbp/p300-interacting transactivator 2	33%	40%	0%
C-type lectin domain family 9 member A	33%	20%	25%
Dickkopf-related protein 2	33%	40%	0%
Homeobox protein BarH-like 1	33%	20%	25%
Homeobox protein MOX-1	33%	40%	0%
Homeobox protein notochord	50%	20%	0%
Intestine-specific homeobox	33%	40%	0%

Mannose-binding protein C	33%	20%	25%
MyoD family inhibitor	33%	40%	0%
Proline-rich protein 23C	50%	20%	0%
Protein disulfide isomerase CRELD2	33%	20%	25%
Protein lifeguard 4	33%	20%	25%
Protein PBMUCL2	33%	40%	0%
Protein YIPF5	33%	40%	0%
Putative methyltransferase-like protein 7A	33%	40%	0%
Putative serine/threonine-protein kinase PRKY	50%	0%	25%
rRNA methyltransferase 2, mitochondrial	17%	40%	25%
Secreted and transmembrane protein 1	33%	40%	0%
Surfeit locus protein 2	33%	40%	0%
Transmembrane protein 70, mitochondrial	33%	40%	0%
Acyl-coenzyme A diphosphatase FITM2	33%	20%	0%
AN1-type zinc finger protein 5	17%	20%	25%
Axonemal dynein light intermediate polypeptide 1	17%	20%	25%
BRI3-binding protein	17%	20%	25%
Cell Division Cycle Associated 5	0%	40%	25%
Chymotrypsin-like elastase family member 1	33%	20%	0%
Claudin-18	17%	20%	25%
C-type lectin domain family 2 member	17%	20%	25%
Follistatin-related protein 3	17%	20%	25%
Granzyme A	33%	20%	0%
HUWE1 Associated Protein Modifying Stress Responses	17%	40%	0%
Keratin-associated protein 10-2	33%	20%	0%
Killer Cell Lectin Like Receptor C1	17%	40%	0%
Ly6/PLAUR domain-containing protein 3	33%	20%	0%
Lysozyme g-like protein 2	33%	20%	0%
Myelin peripheral protein	17%	20%	25%
PABIR family member 2	17%	20%	25%
Peptidyl-prolyl cis-trans isomerase F, mitochondrial	17%	20%	25%
Potassium Channel Tetramerization Domain Containing 2	33%	20%	0%
RING finger protein 175	33%	20%	0%

Serine protease 1	33%	20%	0%
Synapse differentiation-inducing gene protein 1-like	33%	20%	0%
Transmembrane emp24 domain-containing protein 6	33%	20%	0%
Adenylate kinase 2, mitochondrial	17%	20%	0%
ARL14 effector protein	0%	20%	25%
CD40 ligand	17%	20%	0%
Claudin-12	0%	20%	25%
COMM domain-containing protein 5	0%	20%	25%
Corrinoid adenosyltransferase MMAB	17%	0%	25%
Cytochrome c oxidase assembly protein COX11, mitochondrial	17%	20%	0%
Epididymal-specific lipocalin-10	17%	0%	25%
ER membrane protein complex subunit 3	17%	20%	0%
Four and a half LIM domains protein 3	33%	0%	0%
Glycogenin-1	17%	20%	0%
Homeobox protein Nkx-2.8	17%	20%	0%
Insulin-like growth factor-binding protein 1	17%	20%	0%
Kallikrein-8	17%	20%	0%
Keratin-associated protein 10-8	17%	20%	0%
Lactosylceramide 4-alpha-galactosyltransferase	17%	0%	25%
Ly6/PLAUR domain-containing protein 5	17%	20%	0%
Mannose-P-dolichol utilization defect 1 protein	0%	20%	25%
Myb/SANT-like DNA-binding domain-containing protein 1	33%	0%	0%
Neuroblastoma suppressor of tumorigenicity 1	17%	20%	0%
Olfactory receptor 12D2	0%	20%	25%
Olfactory receptor 1E1	0%	20%	25%
Olfactory receptor 2L2	0%	20%	25%
Potassium Channel Tetramerization Domain Containing 6	17%	20%	0%
Proteasome subunit alpha type-6	0%	20%	25%
Protein Wnt-3a	17%	0%	25%
Protein YIPF4	0%	20%	25%
Putative cytosolic acyl coenzyme A thioester hydrolase-like	17%	0%	25%
Ras-like protein family member 11A	17%	20%	0%
Regulatory Factor X Associated Ankyrin Containing Protein	17%	20%	0%

Ring Finger Protein 125	0%	20%	25%
RINGO Cell Cycle Regulator Family Member E4	0%	20%	25%
Sperm acrosome membrane-associated protein 3	0%	20%	25%
TCF3 fusion partner	0%	20%	25%
Tetraspanin-3	17%	20%	0%
Tribbles Pseudokinase 2	0%	20%	25%
Uncharacterized protein C17orf50	33%	0%	0%
Uncharacterized protein C22orf46	0%	20%	25%
Vascular endothelial growth factor D	17%	0%	25%
Zinc finger FYVE domain-containing protein 21	17%	20%	0%

[a] T1 – tumor size <3 cm
[b] T2 – tumor size >3 but <7 cm
[c] T3+T4 – tumor size >7 cm

Table S6. Frequency of proteins identified by LC-MS analysis only in NSCLC patients according to N feature (pTNM classification).

**Protein name**	**NSCLC patients with N0 feature[a]**	**NSCLC patients with N1 feature[b]**	**NSCLC patients with N2 feature[c]**
Olfactory receptor 2AT4	100%	67%	75%
Olfactory receptor 9G9	80%	83%	75%
Homeobox protein DBX1	80%	67%	75%
R-spondin-2	100%	50%	75%
Killer cell immunoglobulin-like receptor 2DL1	80%	67%	25%
Olfactory receptor 52R1	80%	67%	50%
Gamma-interferon-inducible lysosomal thiol reductase	80%	33%	75%
Neurexophilin-3	80%	33%	100%

Olfactory receptor 13C4	60%	50%	75%
Olfactory receptor 2T4	60%	67%	25%
Protein FAM166B	40%	50%	100%
Inactive phospholipid phosphatase 7	60%	50%	50%
Myeloid leukemia factor 2	60%	33%	75%
Pulmonary surfactant-associated protein A2	60%	33%	75%
Thioredoxin-dependent peroxide reductase, mitochondrial	60%	50%	50%
Transcription factor JunB	60%	50%	50%
Activator of basal transcription 1	60%	33%	50%
Alkaline ceramidase 1	60%	33%	50%
BPI fold-containing family A member 3	60%	33%	50%
Chymotrypsin-like elastase family member 2B	60%	33%	50%
Dysbindin domain-containing protein 2	60%	33%	50%
EF-hand domain-containing protein D1	60%	17%	75%
GS homeobox 1	60%	17%	50%
HLA class II histocompatibility antigen, DM alpha chain	60%	33%	50%
HLA class II histocompatibility antigen, DO beta chain	60%	33%	50%
Homeobox protein Hox-C4	60%	33%	50%
Inactive Rho GTPase-activating protein 11B	60%	33%	50%
Low-density lipoprotein receptor class A domain-containing protein 2	60%	33%	50%
Multivesicular body subunit 12A	60%	33%	50%
Out at first protein homolog	60%	33%	50%
Phosphoglycerate mutase 2	60%	17%	75%
Protein odd-skipped-related 1	60%	33%	75%
Putative transcription factor Ovo-like 1	60%	33%	50%
SPRY domain-containing SOCS box protein 4	60%	33%	50%
Transcription factor Ovo-like 2	60%	33%	50%
Transcription factor PU.1	60%	33%	50%
Transmembrane protein 187	60%	33%	50%
Transmembrane protein 51	60%	17%	75%
Uroplakin-1b	60%	17%	50%
Acrosomal protein SP-10	60%	17%	50%
GATA zinc finger domain-containing protein 1	40%	33%	50%

Pleckstrin homology domain-containing family F member 1	40%	33%	50%
Protein lyl-1	60%	33%	25%
Synapse differentiation-inducing gene protein 1	60%	33%	25%
Cdc42 effector protein 3	20%	17%	75%
Claudin-15	20%	33%	50%
Embryonic polyadenylate-binding protein 2	60%	17%	25%
Erlin-1	60%	17%	25%
GTP cyclohydrolase 1	20%	17%	75%
Inhibin beta C chain	60%	17%	25%
Ly6/PLAUR domain-containing protein 8	60%	17%	25%
Mitochondrial import inner membrane translocase subunit Tim29	40%	33%	25%
Peptidase inhibitor R3HDML	40%	17%	50%
Phosphomannomutase 1	60%	17%	25%
Protein BEAN1	40%	33%	25%
Protein FAM210A	60%	17%	25%
Putative uncharacterized protein FLJ37218	60%	17%	25%
Ras association domain-containing protein 3	40%	17%	50%
Ras-related protein Rab-40A	40%	33%	25%
Receptor expression-enhancing protein 2	20%	17%	75%
Transmembrane protein 106B	60%	0%	50%
Amphiregulin	40%	17%	25%
Cathepsin B	40%	17%	25%
Cation-dependent mannose-6-phosphate receptor	20%	33%	25%
Cbp/p300-interacting transactivator 2	40%	0%	50%
C-type lectin domain family 9 member A	20%	17%	50%
Dickkopf-related protein 2	40%	17%	25%
Follistatin-related protein 3	40%	33%	0%
Homeobox protein BarH-like 1	40%	0%	50%
Homeobox protein MOX-1	40%	17%	25%
Homeobox protein notochord	20%	33%	25%
Interleukin-34	40%	17%	50%
Intestine-specific homeobox	40%	17%	25%
Ly6/PLAUR domain-containing protein 3	40%	17%	0%

Mannose-binding protein C	40%	0%	50%
MyoD family inhibitor	40%	17%	25%
Proline-rich protein 23C	40%	33%	0%
Protein disulfide isomerase CRELD2	60%	17%	0%
Protein lifeguard 4	20%	17%	50%
Protein PBMUCL2	40%	17%	25%
Protein YIPF5	40%	17%	25%
Putative methyltransferase-like protein 7A	40%	17%	25%
Putative serine/threonine-protein kinase PRKY	20%	17%	50%
RING finger protein 175	0%	50%	0%
rRNA methyltransferase 2, mitochondrial	40%	0%	50%
Secreted and transmembrane protein 1	40%	17%	25%
Surfeit locus protein 2	20%	33%	25%
Thiol S-methyltransferase METTL7B	40%	0%	50%
Transmembrane protein 70, mitochondrial	40%	17%	25%
Very-long-chain (3R)-3-hydroxyacyl-CoA dehydratase 2	40%	0%	50%
Acyl-coenzyme A diphosphatase FITM2	40%	17%	0%
AN1-type zinc finger protein 5	20%	0%	50%
Axonemal dynein light intermediate polypeptide 1	40%	17%	0%
BRI3-binding protein	20%	0%	50%
BTB/POZ domain-containing protein KCTD2	20%	33%	0%
Chymotrypsin-like elastase family member 1	40%	17%	0%
Claudin-18	40%	17%	0%
C-type lectin domain family 2 member L	20%	0%	50%
Granzyme A	40%	17%	0%
Keratin-associated protein 10-2	20%	17%	25%
Lysozyme g-like protein 2	20%	0%	50%
Myelin protein P0	20%	17%	25%
NKG2-A/NKG2-B type II integral membrane protein	20%	0%	50%
PABIR family member 2	40%	17%	0%
Peptidyl-prolyl cis-trans isomerase F, mitochondrial	20%	17%	25%
Serine protease 1	20%	17%	25%
Sororin	40%	0%	25%

Synapse differentiation-inducing gene protein 1-like	20%	17%	25%
Telomere attrition and p53 response 1 protein	20%	0%	50%
Transmembrane emp24 domain-containing protein 6	20%	17%	25%
Speedy protein E4	20%	0%	25%
Adenylate kinase 2, mitochondrial	20%	17%	0%
ARL14 effector protein	40%	0%	0%
BTB/POZ domain-containing protein KCTD6	20%	17%	0%
CD40 ligand	40%	0%	0%
Claudin-12	20%	0%	25%
COMM domain-containing protein 5	20%	0%	25%
Corrinoid adenosyltransferase MMAB	0%	0%	50%
Cytochrome c oxidase assembly protein COX11, mitochondrial	0%	0%	50%
DNA-binding protein RFXANK	20%	0%	25%
E3 ubiquitin-protein ligase RNF125	0%	17%	25%
Epididymal-specific lipocalin-10	20%	17%	0%
ER membrane protein complex subunit 3	40%	0%	0%
Four and a half LIM domains protein 3	0%	17%	25%
Glycogenin-1	0%	33%	0%
Homeobox protein Nkx-2.8	20%	17%	0%
Insulin-like growth factor-binding protein 1	20%	17%	0%
Kallikrein-8	20%	17%	0%
Keratin-associated protein 10-8	20%	0%	25%
Lactosylceramide 4-alpha-galactosyltransferase	40%	0%	0%
Ly6/PLAUR domain-containing protein 5	20%	17%	0%
Mannose-P-dolichol utilization defect 1 protein	40%	0%	0%
Myb/SANT-like DNA-binding domain-containing protein 1	20%	0%	25%
Neuroblastoma suppressor of tumorigenicity 1	0%	33%	0%
Olfactory receptor 12D2	20%	0%	25%
Olfactory receptor 1E1	0%	17%	25%
Olfactory receptor 2L2	0%	17%	25%
Proteasome subunit alpha type-6	40%	0%	0%
Protein Wnt-3a	20%	17%	0%
Protein YIPF4	40%	0%	0%

Putative cytosolic acyl coenzyme A thioester hydrolase-like	0%	0%	50%
Ras-like protein family member 11A	40%	0%	0%
Sperm acrosome membrane-associated protein 3	20%	0%	25%
TCF3 fusion partner	20%	0%	25%
Tetraspanin-3	40%	0%	0%
Tribbles homolog 2	20%	0%	25%
Uncharacterized protein C17orf50	20%	17%	0%
Uncharacterized protein C22orf46	40%	0%	0%
Vascular endothelial growth factor D	40%	0%	0%
Zinc finger FYVE domain-containing protein 21	20%	17%	0%

[a] N0 – no regional node metastasis
[b] N1– metastasis in ipsilateral pulmonary or hilar nodes
[c] N2 – metastasis in ipsilateral mediastinal or subcarinal nodes

Table S7. Qualitative analysis of exosome markers in the serum of NSCLC patients and the control group based on the mean fluorescence intensity (MFI) value obtained in the immunoassay (MAGPIX system).

	**The mean fluorescence intensity (MFI)**		
	**CD9**	**CD81**	**CD63**
**BLANK**	3,3	7,8	3,3
**control 1**	39,5	2	3
**control 2**	6	1	2
**control 3**	121	17	26
**patient 1**	1136,5	51	220,5
**patient 2**	332	2	7
**patient 3**	585	27,5	39,5

**patient 4**	806	38	26,5
**patient 5**	678	19	74
**patient 6**	787	36	81
**patient 7**	331	21	64,5
**patient 8**	201	21	54
**patient 10**	266	54	44,5
**patient 11**	244,5	467	22
**patient 12**	1802	40	218
**patient 13**	1545,5	308	185
**patient 14**	759,5	25	75
